# Complement, but Not Platelets, Plays a Pivotal Role in the Outcome of Mucormycosis In Vivo

**DOI:** 10.3390/jof9020162

**Published:** 2023-01-25

**Authors:** Verena Harpf, Günter Rambach, Nadia Parth, Magdalena Neurauter, Verena Fleischer, Michaela Lackner, Cornelia Lass-Flörl, Reinhard Würzner, Cornelia Speth

**Affiliations:** 1 Institute of Hygiene and Medical Microbiology, Medical University of Innsbruck, 6020 Innsbruck, Austria; 2 Christian Doppler Laboratory for Invasive Fungal Infections, 6020 Innsbruck, Austria

**Keywords:** mucormycetes, complement, C1q, C3, C5b-9, murine model, *Rhizopus*, *Lichtheimia*, *Rhizomucor*, *Mucor*

## Abstract

Background: Mucormycetes, a heterogeneous group of fungi, induce a life-threatening disease called mucormycosis. Immune deficiencies represent a major risk factor; hence, we wanted to illuminate the role of complement and platelets in the defense against mucormycetes. Methods: *Rhizopus arrhizus* (*Ra*), *Rhizopus microsporus* (*Rm*), *Lichtheimia ramosa* (*Lr*), *Lichtheimia corymbifera* (*Lc*), *Rhizomucor pusillus* (*Rmp*), and *Mucor circinelloides* (*Mc*) spores were opsonized with human and mouse serum, and C1q, C3c, and terminal complement complex (C5b-9) deposition was measured. Additionally, thrombocytopenic, C3-deficient, or C6-deficient mice were intravenously infected with selected isolates. Survival and immunological parameters were monitored, and fungal burden was determined and compared to that of immunocompetent and neutropenic mice. Results: In vitro experiments showed significant differences in complement deposition between mucormycetes. *Mc* isolates bound up to threefold more human C5b-9 than other mucormycetes. *Lr*, *Lc*, and *Mc* bound high levels of murine C3c, whereas human C3c deposition was reduced on *Mc* compared to *Lr* and *Lc*. Murine C3c deposition negatively correlated with virulence. Complement deficiencies and neutropenia, but not thrombocytopenia, were shown to be a risk factor for a lethal outcome. Conclusion: Complement deposition varies between mucormycetes. Additionally, we demonstrated that complement and neutrophilic granulocytes, but not platelets, play an important role in a murine model of disseminated mucormycosis.

## 1. Introduction

Mucormycetes are an ancient but rather heterogeneous group of higher fungi belonging to the subphylum of Mucormycotina [1]. These fungi are found worldwide in soil and on rotten plant material as saprobes, but some species of the order Mucorales also act as opportunistic pathogens and, therefore, can cause a disease called mucormycosis [2,3] In Europe, the main etiological agents are *Rhizopus* species (*R. arrhizus*, *R. microsporus*), *Lichtheimia* species (*L. corymbifera*), and *Mucor species* (*M. circinelloides*, *Rhizomucor pusillus*) [4]. Infections due to mucormycetes typically spread rapidly, which can be attributed to the fast growth of these organisms [5]. In addition, angioinvasion, which subsequently leads to thrombosis and ischemic tissue necrosis, is a hallmark of mucormycosis [5]. Depending on the clinical manifestation of the infection, mucormycosis can be classified into different categories, namely, rhinocerebral, cutaneous, pulmonary, gastrointestinal, and disseminated [6]. The prevalence of patients suffering from mucormycosis has increased in the last two decades, mainly due to an increased proportion of the population living with risk factors [7,8]. Patients with hematological malignancies, transplantation, neutropenia, poorly controlled diabetes mellitus, steroid therapy, iron overload, and major trauma are at higher risk for mucormycosis [1,7]. Reversion of underlying risk factors, if possible, is, along with surgical debridement of affected tissue and appropriate antifungal therapy, the state-of-the-art treatment to rescue the patient [9]. The intrinsic drug resistance of mucormycetes against short-tailed azoles and echinocandins limits treatment options [10]. Nevertheless, even if treated promptly and adequately with polyenes, mid-, or long-tailed azoles, mucormycosis is associated with a high mortality, exceeding 60% depending on the site of infection and underlying risk factors [9].

A central part of the first-line endogenous defense against fungal infections such as mucormycosis is the innate immunity [11]. By bridging the innate and the adaptive immune defense, the complement (C) system, comprising more than 40 proteins, soluble plasma factors, cell-associated regulator molecules, and receptors, represents a pivotal player in innate immunity [12]. Different substances trigger the three activation pathways, namely, the classical, lectin, and alternative pathways. Although triggered by different structures, these activation pathways merge in the enzymatic activation and cleavage of complement protein C3 into C3a and C3b [13]. As an anaphylatoxin, C3a bears chemotactic activity towards a variety of cells and has pro-inflammatory properties. The complement fragment C3b, on the other hand, acts as an opsonizing molecule and stimulates the internalization and, therefore, the elimination of pathogens by interaction with receptors on phagocytes [13]. The phagocytosed material is furthermore presented on the cell surface, which initiates an adaptive immune response [14]. Additionally, C3b participates in further steps of the complement cascade, which leads to the cleavage of C5 and, subsequently, to the generation of a terminal complement complex (TCC, C5b-9) [14]. Complement partakes in the clearance of immune complexes, apoptotic and necrotic cells, and cellular debris, cell activation and chemotaxis, and stimulation of the immune network [13].

One part of the immune network that is activated by complement is platelets [15]. Formerly thought to only play an important role in maintaining circulatory system homeostasis by forming thrombi, these small (2–5 µm), circulating, anucleate cell fragments have been shown to also act as multifaceted elements of innate immunity [15,16]. The complement system can activate these cell fragments in various ways: interaction with opsonized bacteria, mediating their clearance [17], binding of C3a to its receptor, increasing platelet adhesion to fibrinogen and increasing platelet aggregation [18], C5a receptor/C5a interaction, inhibiting endothelial cell functions necessary for angiogenesis [19], and insertion of C5b-9 in the platelet membrane, triggering shedding of microparticles [20]. In addition to being activated by the complement system, platelets can activate the complement system via the classical pathway, binding complement components such as C1q and C4d, and via the alternative pathway, mediating the spontaneous hydrolysis of C3 [15]. In addition to complement interactions, platelets react upon the interaction of various pathogens with their toll-like receptors and sense immune complexes via FcγRIIa and activate T cells and mediate antigen-presenting cell differentiation [15].

A feature of mucormycosis is angioinvasion, where the fungus comes into close contact with platelets, which were shown previously to exert antifungal properties toward mucormycetes in vitro [21]. Moreover, platelets are known to interact with the complement system, and immunocompromised individuals are at higher risk of suffering from mucormycosis. For these reasons, the aim of this study was to examine the role of complement, platelets, and their interplay within the antimicrobial host defense system in a murine model of disseminated mucormycosis.

## 2. Materials and Methods

### 2.1. Reagents and Media

Glucose, ammonium chloride (NH_4_Cl), magnesium sulfate heptahydrate (MgSO_4_ × 7H_2_O), potassium dihydrogenphosphate (KH_2_PO_4_), dipotassium phosphate (K_2_HPO_4_), formaldehyde solution 37%, calcium chloride (CaCl_2_), ethylenediaminetetraacetic acid (EDTA), tetramethylbenzidine (TMB), sodium acetate (C_2_H_3_NaO_2_), hydrogen peroxide (H_2_O_2_) 30%, and sulfuric acid (H_2_SO_4_) 96% were purchased from Carl Roth (Karlsruhe, Germany). Tween20, yeast extract, disodium phosphate dihydrate (Na_2_HPO_4_ × 2H_2_O), bovine serum albumin (BSA), and dimethyl sulfoxide anhydrous (DMSO) ≥ 99.9% were obtained from Sigma Aldrich (St. Louis, MO, USA). Sodium chloride (NaCl), sodium dihydrogen phosphate monohydrate (NaH_2_PO_4_ × H_2_O), and magnesium chloride hexahydrate (MgCl_2_ × 6H_2_O) were bought from MERCK (Darmstadt, Germany). Agar was purchased from VWR International (Radnor, PA, USA). Rat anti-mouse sCD62P capture antibody, rat anti-mouse sCD62P detection antibody, sCD62P standard, and horseradish peroxidase-conjugated streptavidin were ordered from R&D Systems, Inc. (Minneapolis, MN, USA). Fluorescein isothiocyanate (FITC)-conjugated rabbit anti-human C3c antibody and rabbit anti-human C1q antibody were supplied by Agilent Technologies (Santa Clara, CA, USA). FITC-conjugated mouse anti-human TCC antibody reacting with a C9 neoantigen was obtained from Hycult (Wayne, PA, USA). Alexa Fluor© 488-conjugated goat anti-rabbit antibody was provided by Life Technologies (Carlsbad, CA, USA). Rat anti-mouse Ly6G antibody was acquired from Biozol Diagnostica GmbH (Eching, Germany). Rabbit anti-mouse platelet serum was purchased from Cedarlane Laboratories (Burlington, Ontario, Canada). Rat anti-mouse CD41 antibody was obtained from BioLegend (San Diego, CA, USA).

### 2.2. Fungal Isolates and Cultivation

The strains listed in Table S1 are environmental or patient isolates, and several of these were confirmed by sequencing the internal transcribed spacer (ITS) region of the ribosomal genes [22]. Isolates were grown on SUP (supplemented minimal medium for mucormycetes [23]) agar at either 30 °C for *M. circinelloides* isolates or 37 °C for isolates from other mucormycetes until sporulation was visible. Sporangiospores were harvested in sterile spore buffer (PBS with 0.05% Tween20), filtered through a 40 µm cell strainer (BD Diagnostic System, Heidelberg, Germany), and washed with either 0.9% NaCl for the animal experiment or PBS for the in vitro experiments. For the animal experiments, the suspension was further filtered using a 10 µm cell strainer (Sysmex Europe SE, Norderstedt, Germany). The spore suspension was counted with a hemocytometer and either diluted in 0.9% NaCl for immediate use in the murine model or in PBS for in vitro experiments. Stock suspensions were stored at 4 °C for up to 3 months.

### 2.3. Human and Murine C1q and C3c Deposition and Human C5b-9 Deposition on Mucormycete Spores

Stock suspensions of the strains listed in Table S1 were prepared as described above. Five hundred thousand spores of each isolate were opsonized for either 60 min (C1q or C3c deposition) or 90 min (C5b-9 deposition) at 37 °C with either 60% normal human serum (NHS) from an NHS pool or 60% normal mouse serum (NMS) from an NMS pool. Fifty percent (*v*/*v*_NHS/NMS_) of PBS++ (0.15 mM CaCl_2_ and 1 mM MgCl_2_ in PBS) was added to supply the sufficient number of ions necessary for the activation of the complement system. As a negative control, murine or human serum was replaced by heat-inactivated serum, which was obtained by heating the serum for 30 min at 60 °C in a water bath. Opsonized spores and controls were fixed with PBS/1% formalin for 30 min at room temperature (RT). After fixing, samples were washed with PBS, and unspecific binding sites were blocked for 30 min at RT using PBS/1% BSA. To detect human and murine C1q deposition, 17.75 µg/mL rabbit anti-human C1q antibody was added directly to the samples. For human and murine C3c detection, 7.5 ng/mL FITC-conjugated rabbit anti-human C3c antibody was used, and, to detect human C5b-9, 2.5 µg/mL FITC-conjugated mouse anti-human TCC antibody was added. The samples were incubated for 30 min at RT. After the incubation period, the samples were washed with PBS, and the pellet for the C3c and C5b-9 deposition was resuspended in PBS/1% formalin and fixed for 30 min at RT. For the C1q deposition, the pellet was resuspended in 10 µg/mL Alexa Fluor© 488-conjugated goat anti-rabbit antibody and incubated for 30 min at RT. After the incubation period, the spores were washed with PBS, and the pellet was resuspended in PBS/1% formalin, fixed for 30 min at RT, and measured.

The deposition of human C1q, C3c, and C5b-9 was measured independently using FACS Verse and BD software (BD Bioscience, San Diego, CA, USA). Mucormycete spores were gated by SSC-A vs. FSC-A density plot. Ten thousand gate events were analyzed. The positive population was detected using a one-color density plot SSC-A vs. FITC-A. For the human C1q, C3c, and C5b-9 deposition, the mean fluorescence intensity (MFI) of each strain was normalized against the MFI of RmP1.

### 2.4. Animal Experiments

#### 2.4.1. Ethics Statement

The Central Laboratory Animal Facility of Medical University of Innsbruck and all experimental procedures of the study complied with the Austrian Animal Experimental Act (BGBl. I Nr. 114/2012). All animal experiments were approved by the National Committee for Animal Care of the Austrian Federal Ministry of Science, Research and Economy (BMWFW) (approval numbers BMWFW-66.011/0087-WF/V/3b/2017, BMWFW-66.011/0176-WF/V/3b/2017). Blood sampling and euthanasia of mice were performed under isoflurane anesthesia, and all efforts were made to minimize suffering.

#### 2.4.2. Mouse Lines, Experimental Groups, Humane Endpoints

Seven-week-old C57Bl/6J mice were purchased from Charles River Laboratory (Sulzfeld, Germany). The C3-deficient (ΔC3) and C6-deficient (ΔC6) mice were genetically modified C57Bl/6 mice and were bred at the Institute of Hygiene and Medical Microbiology in Innsbruck. Experimental groups each included nine mice.

Each mouse meeting one of the defined humane endpoints was immediately anesthetized and euthanized by cervical dislocation.

#### 2.4.3. Performance of Animal Experiments

To compare the relevance of single innate immune elements, complement deficiencies at different stages in the cascade were simulated by using either C3-deficient or C6-deficient mice. These mice lacked either the central C3 protein or the terminal C6 protein. Neutropenia was induced according to Bruhn and coworkers [24] by administering a monoclonal rat anti-mouse Ly6G antibody (clone 1A8). The depletion via this blocking antibody led to a 90% reduction of neutrophil numbers for at least 3 days after each injection. Thrombocytopenia was caused by administering a rabbit anti-mouse platelet serum every second day throughout the experiment; platelet numbers were decreased by more than 80%.

At day 0, mice were intravenously infected in the lateral tail vein with 5 × 10^5^ spores of *R. arrhizus* (isolate RO7, CBS 126971), *R. microsporus* (isolate RM9, CBS 102277), *L. ramosa* (isolate LR5, CBS 101.55), *L. corymbifera* (isolate LC9, CBS 109940), *Rh. pusillus* (isolate RmP5, CBS 219.31), or *M. circinelloides* (isolate M6, CBS 394.68). Body weight and body temperature (non-contact surface thermometer, Geratherm) were determined twice a day. A clinical assessment was performed at least twice a day (up to two-hourly intervals for symptomatic animals) after the infection over a period of 14 days. Blood and urine samples were taken at days 1 and 7 after the infection and at the day of exitus, which was either day 14 post infection or whenever an animal met humane endpoints.

#### 2.4.4. Analysis of Blood and Organ Samples

At indicated time points after the infection, EDTA whole blood (100–150 µL) was taken from the submandibular vein of the animals. A blood cell counter (animal blood counter Vet abc, Scil) was used to obtain hemograms. The fungal load in blood was determined by plating 10 µL of whole blood diluted 1:10 in 0.9% NaCl on SUP agar and incubating the Petri dishes for 48 h at 30 °C for *M. circinelloides* or 37 °C for the other mucormycetes used to infect the animals. Residual whole blood was centrifuged at 1377× *g* for 15 min to obtain platelet-poor plasma, which was frozen and stored at −80 °C until ELISA was performed.

After the sacrification of the animals, selected organs (spleen, kidney, liver, lung, and brain) were dissected, weighed, and examined macroscopically. The majority of the organs were frozen in liquid nitrogen and stored at −20 °C until qPCR was performed. Selected organs with macroscopic changes such as kidneys, stomach, and intestine were fixed in PBS/4% formalin for histological examination. Sections of these organs were dyed with a combined hematoxylin and eosin/methenamine silver stain to visualize fungal infestation [25].

### 2.5. Detection of Mucormycetes in the Kidney by Polymerase Chain Reaction

Mechanical lysis, DNA extraction, and qPCR were performed, and standard curves were established as described in our previous work [26].

### 2.6. DuoSet Sandwich Enzyme-Linked Immunosorbent Assay (ELISA) to Detect Murine Soluble P-Selectin/sCD62P in Plasma

Plasma sCD62P levels were measured by quantitative sandwich enzyme immunoassay (R&D Systems, Inc. DY737, Minneapolis, MN, USA).

#### 2.6.1. Plate Preparation

The capture antibody was diluted to the working concentration according to the manufacturer’s protocol. Fifty microliter capture antibody per well was used to coat the 96-well microplate (Greiner Bio-One 655061, Kremsmünster, AUT) and was incubated overnight at RT. Succeeding the incubation, the wells were aspirated and washed three times with 150 µL wash buffer (PBS/0.05% Tween20, pH 7.2–7.4). After the last wash, any remaining liquid was removed by blotting the plate against clean paper towels. Unspecific binding sites were blocked by adding 150 µL reagent diluent (PBS/1% BSA, pH 7.2–7.4) per well and incubating and slightly shaking for 60 min at RT. At the end of the blocking, the plates were washed as described above.

#### 2.6.2. Assay Procedure

The assay was performed according to the manufacturer’s protocol with the following modifications. The volumes of the reagents used were reduced to half, except the volume of the stop solution; standards were diluted in PBS; samples were diluted 1:200 in PBS; the incubation period of the detection antibody was reduced to 60 min; and the incubation of the substrate solution was dependent on the standard well containing 250 pg/mL turning slightly blue. Optical density was determined using a plate reader (Model 680, Bio-Rad, Hercules, CA, USA) set to 450 nm, and the wavelength correction was set to 550 nm. The average zero standard optical density was subtracted from the mean value of the duplicate readings of either standards or samples. A standard curve was calculated utilizing a computer-generated four-parameter logistic (4-PL) curve fit. Concentrations of serum sCD62P were expressed as ng per mL per 10^8^ platelets to normalize the measurements to different platelet counts.

### 2.7. Statistical Analyses

Data obtained were analyzed and plotted using GraphPad Prism 8. Samples obtained from mice were either used in duplicate (sCD62P ELISA) or triplicate (mucormycetes PCR). For further analyses, the means of these values were used. In vitro experiments were performed 3 times on different days. To show significant differences, one-way ANOVAs were performed, and Tukey post hoc analyses were used for further analyzing differences between the different groups with normally distributed values. For skewed distributed values, Kruskal–Wallis tests were performed, and Dunn’s post hoc analyses were used to further analyze the differences between the different groups. The survival curves were analyzed using the Mantel–Cox test. The reported *p*-values throughout the manuscript refer to adjusted *p*-values. The threshold for statistical significance was *p* < 0.05. 

## 3. Results

### 3.1. C1q, C3c, and C5b-9 Deposition on Spores

**Figure 1:**
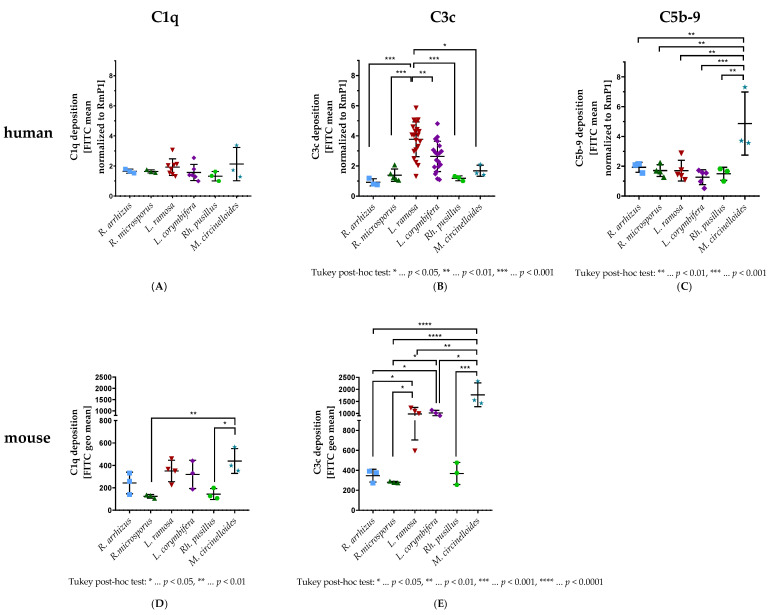
C1q, C3c, and C5b-9 deposition on the spore surface of different strains of *R. arrhizus*, *R. microsporus*, *L. ramosa*, *L. corymbifera*, *Rh. pusillus*, and *M. circinelloides*. Spores were opsonized with 60% of either normal human serum (NHS) (**A**–**C**) or normal mouse serum (NMS) (**D**,**E**) for either 60 min (C1q and C3c) or 90 min (C5b-9) at 37 °C in separate tubes. Specific anti C1q, C3c, or C5b-9 antibodies were used, and the deposition was measured using fluorescence-activated cell sampler (FACS) Verse with BD software. The deposition of human (**A**) and murine (**D**) C1q, human (**B**) and murine (**E**) C3c, and human C5b-9 (**C**) is shown as fluorescein isothiocyanate (FITC) mean normalized to a *Rh. pusillus* isolate (RmP1) (**A**–**C**) or FITC geometric mean (geo mean) (**D**,**E**). The results shown were gained by a minimum of two independently performed experiments, and at least three different strains per species were tested (mean ± SD). Asterisks (*) indicate the level of significance for an adjusted *p*-value, comparing pairs of groups using a Tukey post hoc test after the one-way ANOVA reported significance.

The circulating pattern recognition molecule C1q initiates the complement system via the classical pathway [14]. When incubated with human serum as a source of complement proteins, all tested mucormycetes showed low levels of C1q binding, as measured by a rabbit anti-human C1q antibody that cross-reacts with murine C1q. The amount of human C1q on the surface of the spores did not differ significantly between the various mucormycetes, but a tendency towards higher C1q on *M. circinelloides* was observed (Figure 1A). Mucormycetes incubated with mouse serum exhibited a similar C1q binding pattern (Figure 1D), which was found to be more pronounced than for human C1q (Figure 1A). *Mucor circinelloides* isolates bound significantly more murine C1q compared to *R. microsporus* (315.5, 95% CI (68.6, 562.3), *p* < 0.01) and *Rh. pusillus* (295.1, 95% CI (48.23, 542.0), *p* < 0.05) (Figure 1D). *Lichtheimia corymbifera* and *L. ramosa* showed a tendency towards higher murine C1q deposition on their surface compared to the tested *Rhizopus* and *Rhizomucor* isolates (Figure 1D).

The three activation pathways merge on the level of C3. The cleavage products C3b and C3c can bind to microorganisms and, therefore, represent a central part of opsonizing and, thereby, defeating pathogens [13]. The murine C3c deposition pattern on the mucormycetes (Figure 1E), as measured by a rabbit anti-human C3c antibody exhibiting cross-reactivity with murine C3c, resembled the murine C1q deposition pattern (Figure 1D). *Mucor circinelloides* isolates showed the highest amount of C3c on their surface, which was found to be significantly elevated compared to all other species. Moderate murine C3c levels were found on *Lichtheimia* spp., which were significantly higher than the ones found for *Rhizopus* species (Figure 1E). In contrast to the murine C3c deposition pattern, the human C3c deposition pattern (Figure 1B) differed from the human C1q deposition pattern (Figure 1A), as *M. circinelloides* bound only minimal amounts of C3c. *Lichtheimia ramosa* bound significantly more C3c than all other tested species (Figure 1B).

The terminal complement complex (TCC, C5b-9) is the final product of the complement cascade, which can either bind to cells, lysing them, or, in its soluble form, lead to further immune cell activation [14]. C5b-9 was found to bind on all tested mucormycetes (Figure 1C). The C5b-9 amounts differed significantly (2–4× in amount; F(5, 17) = 7.568, *p* < 0.001) for *M. circinelloides* compared to all other mucormycetes tested (Figure 1C).

### 3.2. The Virulence of Selected Mucormycetes Negatively Correlated with the C3c Deposition on Spores

Since we showed that the complement deposition, especially opsonization, on different mucormycetes varies in vitro, we investigated whether this translates to differences in virulence in immunocompetent animals.

**Figure 2:**
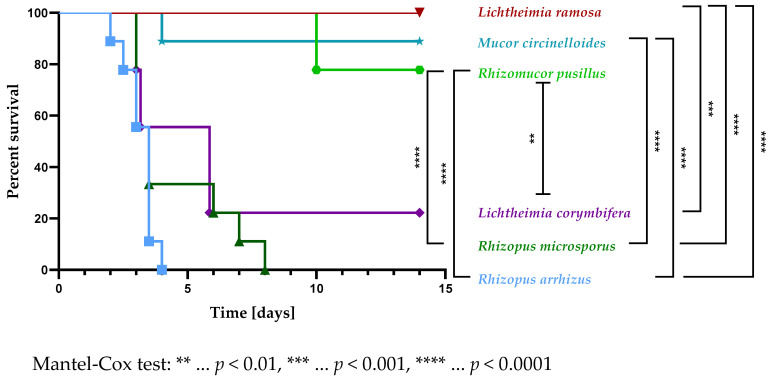
Survival curves in a murine model of disseminated mucormycosis. Immunocompetent (wt, *n* = 9) C57Bl/6J mice were intravenously infected with 5 × 10^5^ spores of *R. arrhizus*, *R. microsporus*, *L. ramosa*, *L. corymbifera*, *Rh. pusillus*, or *M. circinelloides*. The survival was monitored for 14 days. Asterisks (*) indicate the level of significance for a *p*-value resulting from a Mantel–Cox test analyzing pairs of survival curves.

When intravenously infected with 5 × 10^5^ spores of *R. arrhizus*, *R. microsporus*, *L. ramosa*, *L. corymbifera*, *Rh. pusillus*, or *M. circinelloides*, significant differences in the virulence of the tested isolates were noted in immunocompetent C57Bl/6J mice (Figure 2). When challenged with the least virulent isolate, *L. ramosa*, none of the immunocompetent animals met a humane endpoint throughout the experiment (Figure 2). The isolate was shown to be significantly less virulent than *L. corymbifera* (χ^2^ (1) = 10.86, *p* < 0.01), *R. microsporus* (χ^2^ (1) = 19.78, *p* < 0.0001), and *R. arrhizus* (χ^2^ (1) = 18.03, *p* < 0.0001) (Figure 2). Animals challenged with *R. arrhizus* showed the highest mortality, with a lethality of all immunocompetent animals within four days (Figure 2). *Rhizopus arrhizus* was found to be significantly more virulent than *M. circinelloides* (χ^2^ (1) = 16.73, *p* < 0.0001), *L. ramosa* (χ^2^ (1) = 18.03, *p* < 0.0001), and *Rh. pusillus* (χ^2^ (1) = 18.03, *p* < 0.0001) (Figure 2).

**Figure 3:**
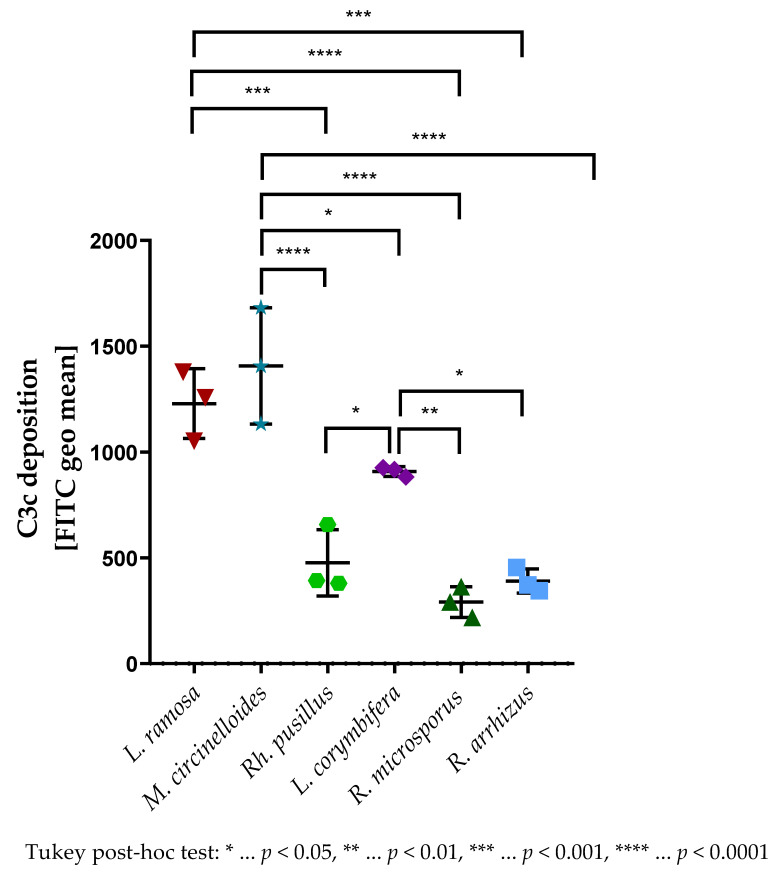
C3c deposition on strains used for the murine model of disseminated mucormycosis. Spores of *R. arrhizus*, *R. microsporus*, *L. ramosa*, *L. corymbifera*, *Rh. pusillus*, or *M. circinelloides* were opsonized with 60% mouse serum for 30 min at 37 °C. A specific anti-C3c antibody was used, and the deposition was measured using fluorescence-activated cell sampler (FACS) Verse with BD software. The deposition of C3c is shown as fluorescein isothiocyanate (FITC) geometric mean (geo mean). The results shown were gained by three independently performed experiments (mean ± SD). Asterisks (*) indicate the level of significance for an adjusted *p*-value, comparing pairs of groups using a Tukey post hoc test after the one-way ANOVA reported significance.

The murine C3c deposition pattern on spores of the tested mucormycetes (Figure 3) was negatively correlated with the virulence of respective isolates in immunocompetent mice (Figure 2). Hence, spores of the least virulent isolates, such as *L. ramosa* and *M. circinelloides*, bound the highest amounts of C3c (Figure 3). In contrast to all other tested isolates, for *Rh. pusillus*, although it showed limited mortality in immunocompetent animals challenged with 5 × 10^5^ spores (Figure 2), significantly lower murine C3c levels were measured compared to for *M. circinelloides* (−930.6, 95% CI (−1344, −517.2), *p* < 0.0001), *L. ramosa* (−752.6, 95% CI (−1166, −339.2), *p* < 0.001), and *L. corymbifera* (−431.8, 95% CI (−845.1, −18.43), *p* < 0.05) (Figure 3).

### 3.3. Absence of Neutrophils, C3, or C6, but Not Platelets, Increased the Lethality in a Murine Model of Disseminated Mucormycosis

It is known that neutropenia and immunodeficiencies due to medication such as corticosteroids or cyclophosphamide represent a major risk factor for mucormycosis [1,7]. Revealing further risk factors for a worse outcome of mucormycosis is essential for a better understanding of the disease. In the conducted study, we examined the effect of thrombocytopenia and complement deficiencies on the virulence of the above-mentioned isolates of *R. arrhizus*, *R. microsporus*, *L. ramosa*, *L. corymbifera*, *Rh. pusillus*, and *M. circinelloides* in a murine model of disseminated mucormycosis.

**Figure 4:**
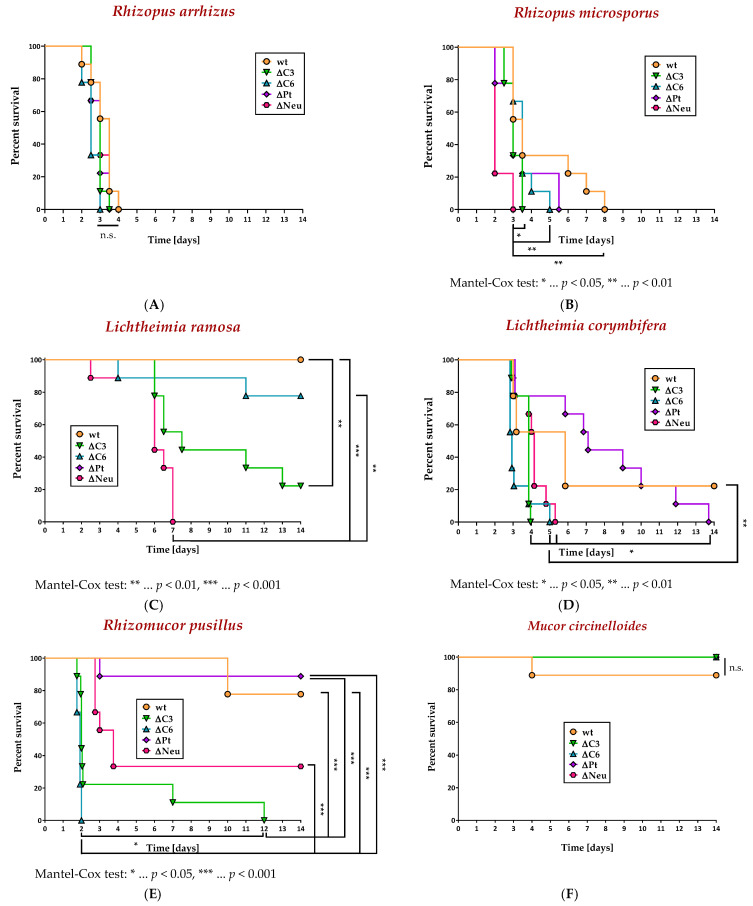
Survival curves in a murine model of disseminated mucormycosis. Immunocompetent (wt, *n* = 9), C3-deficient (ΔC3, *n* = 9), C6-deficient (ΔC6, *n* = 9), thrombocytopenic (ΔPt, *n* = 9), and neutropenic (ΔNeu, *n* = 9) animals were intravenously challenged with 5 × 10^5^ spores of *R. arrhizus* (**A**), *R. microsporus* (**B**), *L. ramosa* (**C**), *L. corymbifera* (**D**), *Rh. pusillus* (**E**), or *M. circinelloides* (**F**). The survival was monitored for 14 days. Asterisks (*) indicate the level of significance for a *p*-value resulting from a Mantel–Cox test analyzing pairs of survival curves.

The chosen dosage of 5 × 10^5^ spores per mouse was found to show no significant differences between immunocompetent and immunodeficient animals when infected with two of the six isolates due to the isolates being either too virulent (*R. arrhizus*, Figure 4A) or not virulent at all (*M. circinelloides*, Figure 4F). In the four residual isolates, neutropenia was confirmed as a risk factor for a lethal outcome in disseminated mucormycosis. The mortality of neutropenic animals was significantly elevated compared to that of immunocompetent individuals when infected with either *R. microsporus* (χ^2^ (1) = 12.31, *p* < 0.01, Figure 4B) or *L. ramosa* (χ^2^ (1) = 17.84, *p* < 0.001, Figure 4C).

In addition to neutropenia, we showed that complement deficiencies in mice contributed to a worsened outcome (Figure 4A–F). This poor outcome (comparative with that of immunocompetent mice) was most pronounced in C6-deficient animals challenged with *L. corymbifera* (χ^2^ (1) = 8.617, *p* < 0.01, Figure 4D) and *Rh. pusillus* (χ^2^ (1) = 18.2, *p* < 0.001, Figure 4E). The mortality (compared to immunocompetent animals) in C3-deficient mice was significantly increased when they were intravenously infected with either *L. ramosa* (χ^2^ (1) = 11.36, *p* < 0.01, Figure 4C) or *Rh. pusillus* (χ^2^ (1) = 16.32, *p* < 0.001, Figure 4E). For infections caused by *Rh. pusillus* and *L. corymbifera*, complement deficiencies of the host led to even higher mortality rates than neutropenia (Figure 4D,E).

As a third immune deficiency, we tested thrombocytopenia. No impact of thrombocytopenia on mortality rates was seen for any of the causative agents of infection (Figure 4A–F).

### 3.4. High Kidney Fungal Load in Complement-Deficient and Neutropenic Animals

After showing that neutrophilic granulocytes and the complement system, but not platelets, play a central role in the outcome of mucormycosis in vivo, we investigated whether the fungal load in tissue samples also differs within the different immunodeficiencies and immunocompetent animals. In a disseminated mucormycosis, kidneys as filter organs are of special interest due to their high perfusion rate. To quantify the genomic fungal DNA in the mice kidneys, we used an in-house PCR based on the translation elongation factor (TEF) of mucormycetes.

**Figure 5:**
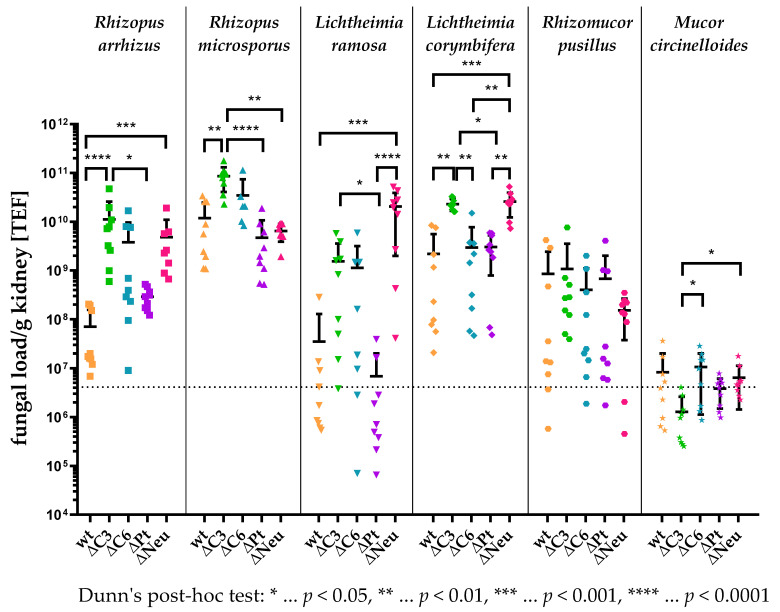
Fungal load per gram kidney tissue. Immunocompetent (wt, *n* = 9), C3-deficient (ΔC3, *n* = 9), C6-deficient (ΔC6, *n* = 9), thrombocytopenic (ΔPt, *n* = 9), and neutropenic (ΔNeu, *n* = 9) animals were intravenously challenged with 5 × 10^5^ spores of *R. arrhizus*, *R. microsporus*, *L. ramosa*, *L. corymbifera*, *Rh. pusillus*, or *M. circinelloides*. Fungal load in the kidney was quantified by PCR based on the translation elongation factor (TEF) of mucormycetes at the day of exitus. Asterisks (*) indicate the level of significance for an adjusted *p*-value, comparing pairs of groups within a fungal species using a Dunn’s post hoc test after the Kruskal–Wallis test reported significance.

The kidneys of immunocompetent animals infected with 5 × 10^5^ spores of *M. circinelloides* were found to have the lowest amount of genomic fungal DNA per gram tissue (Figure 5), which is coherent with the low virulence of this species (Figure 2). Furthermore, the virulence of *L. ramosa*, *Rh. pusillus*, *L. corymbifera*, and *R. microsporus* in immunocompetent animals resembled, in ascending order, the fungal burden in the kidneys of the animals. In contrast, animals challenged with *R. arrhizus* exhibited a low fungal load even though this isolate was shown to be highly virulent throughout all groups (Figure 4A).

In addition, the kidney fungal load of the different immunocompromised groups infected with *M. circinelloides* did not differ significantly from the kidney fungal burden of immunocompetent animals (Figure 5). However, C3-deficient animals were found to have less fungal DNA in their kidneys than C6-deficient (*p* < 0.05) and neutropenic animals (*p* < 0.05). Unlike in the case of those infected with *M. circinelloides*, the survival curves of the animals infected with *Rh. pusillus* (Figure 4E) did not match the fungal load in the kidneys (Figure 5), since the amount of fungal DNA in the tested tissue of immunodeficient animals did not differ significantly compared to that of immunocompetent mice. However, the fungal burden of immunocompetent animals challenged with the four remaining tested isolates differed considerably from the fungal load in immunodeficient mice.

Neutropenia led to significantly elevated fungal burden compared to immunocompetent animals in three out of four species, namely, *R. arrhizus* (*p* < 0.001), *L. ramosa* (*p* < 0.001), and *L. corymbifera* (*p* < 0.001). The amount of fungal DNA measured in neutropenic animals challenged with *R. microsporus* was found to be slightly less compared to immunocompetent animals (Figure 5), although higher lethality in neutropenic animals was observed (Figure 4B).

Although the worst outcome in this murine model was linked to neutropenia, the highest fungal load was measured in C3-deficient animals challenged with *R. microsporus* (Figure 5). Overall, complement-deficient animals exhibited a higher fungal burden compared to immunocompetent animals. This difference was shown as significant by Dunn’s post hoc test for C3-deficient animals infected with either *R. arrhizus* (*p* < 0.0001), *R. microsporus* (*p* < 0.01), or *L. corymbifera* (*p* < 0.01).

The fungal load in the kidneys of thrombocytopenic animals challenged with any of the six tested isolates did not significantly differ compared to immunocompetent animals (Figure 5), which resembled the survival of these animals in this model (Figure 4A–F). Remarkably, thrombocytopenic animals infected with *L. ramosa* exhibited a significantly reduced fungal burden compared to neutropenic (*p* < 0.0001) or C3-deficient (*p* < 0.05) mice. Furthermore, the fungal load of kidneys was significantly elevated in C3-deficient animals compared to thrombocytopenic mice challenged with either *R. arrhizus* (*p* < 0.05), *R. microsporus* (*p* < 0.0001), or *L. corymbifera* (*p* < 0.05).

### 3.5. sCD62P Levels Are Increased in Neutropenic and Complement-Deficient Animals

**Figure 6:**
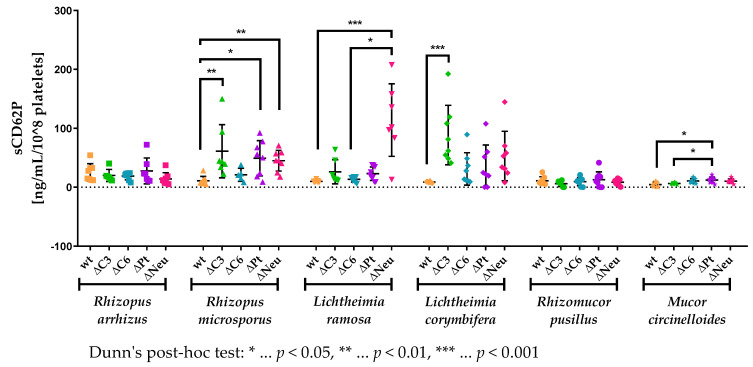
Normalized plasma levels of soluble P-selectin (sCD62P) in a murine model of disseminated mucormycosis. Immunocompetent (wt, *n* = 9), C3-deficient (ΔC3, *n* = 9), C6-deficient (ΔC6, *n* = 9), thrombocytopenic (ΔPt, *n* = 9), and neutropenic (ΔNeu, *n* = 9) animals were intravenously challenged with 5 × 10^5^ spores of *R. arrhizus*, *R. microsporus*, *L. ramosa*, *L. corymbifera*, *Rh. pusillus*, or *M. circinelloides*. Soluble P-selectin levels were measured using serum retrieved from animals at the day of exitus and normalized to the amount of 10^8^ platelets measured at the same time point using whole blood. Asterisks (*) indicate the level of significance for an adjusted *p*-value, comparing pairs of groups within a fungal species using a Dunn’s post-hoc test after the Kruskal–Wallis test reported significance.

A characteristic of mucormycosis is angioinvasion, which is associated with thrombosis [5]. To elucidate the relevance of various immunodeficiencies to platelet activation, we assessed the plasma levels of a biomarker, namely, soluble P-selectin (sCD62P), using a sandwich ELISA in mice intravenously infected with isolates of different mucormycete species (Figure 6). We normalized the sCD62P levels to 1 × 10^8^ platelets of the respective mice to eliminate the variation due to significantly different platelet levels in the tested animals.

In immunocompetent animals, the highest levels of sCD62P, when normalized to the platelet count, were found in mice infected with 5 × 10^5^ spores of *R. arrhizus* and the lowest in *M. circinelloides* (Figure 6), which fitted to the respective virulence (Figure 2). The differences in the normalized platelet activation in immunocompetent mice infected with either one of the residual four strains of mucormycetes were subtle, which was also the case for differences between immunocompetent and immunodeficient animals intravenously infected with either *R. arrhizus* or *Rh. pusillus*. The normalized plasma sCD62P levels of mice challenged with *R. microsporus*, *L. ramosa*, *L. corymbifera*, or *M. circinelloides*, however, did differ considerably between immunocompetent and immunodeficient animals (Figure 6).

Especially, neutropenia led to elevated, normalized sCD62P levels in the plasma of the challenged animals. The performed Dunn’s post hoc test reported differences to be significant in mice intravenously infected with *R. microsporus* (*p* < 0.01) or *L. ramosa* (*p* < 0.001, Figure 6). In addition, the latter isolate exhibited significantly higher murine plasma levels of normalized sCD62P in neutropenic animals compared to in C6-deficient mice (*p* < 0.05).

Although both tested complement deficiencies were demonstrated to have an impact on the survival (Figure 4B–E) and the fungal load in kidneys (Figure 5) of mice challenged with different mucormycetes, C6-deficient animals did not show significantly elevated sCD62P levels compared to immunocompetent mice. In contrast to C6 deficiency, C3 deficiency led to significantly increased platelet activation in mice intravenously infected with either *R. microsporus* (*p* < 0.01) or *L. corymbifera* (*p* < 0.001, Figure 6).

Despite neither showing any effects on the survival (Figure 4A–F) nor significant differences in fungal burden of the kidneys (Figure 5) compared to immunocompetent animals, the levels of soluble P-selectin were elevated in thrombocytopenic animals (Figure 6). The conducted Dunn’s post hoc test reported these differences between thrombocytopenic and immunocompetent mice to be significant when animals were intravenously infected with either *R. microsporus* (*p* < 0.05) or *M. circinelloides* (*p* < 0.05). Furthermore, thrombocytopenic animals challenged with *M. circinelloides* exhibited significantly more platelet activation compared to C3-deficient mice (*p* < 0.05).

## 4. Discussion

Mucormycosis is a devastating disease with a high lethality ranging from 20% to 100%, depending on several factors such as treatment, site of infection, and underlying risk factors [9,27]. Understanding the relevance of different elements of the immune system in pathogenesis is crucial to efficiently tackle this invasive fungal infection. Hence, we investigated the interplay of the complement system and platelets within the antimicrobial host defense system in vitro and in vivo.

In vitro, we checked the deposition of key components of the complement system on mucormycetes. The deposition of C3 and C4 was previously measured on spores of different *Mucor* species opsonized with normal human serum (NHS) and was shown to be of a similar extent on those species [28]. These findings suggest that complement deposition most likely does not differ within a sole species. However, in our study, this was not the case (Figure 1A–E). Especially, the tested isolates of *M. circinelloides* opsonized with normal human serum (NHS) showed considerable differences in C1q and C5b-9 deposition on the spore surface (Figure 1A,C). These variations could be due to the heterogeneity of the *M. circinelloides* complex, which might have been solved by a rather recent taxonomic revision of this complex. The mentioned species revision was shown to be beneficial for dissecting the differences in antifungal susceptibility of the *M. circinelloides* complex [29]. In addition to the differences in C1q deposition within the *M. circinelloides* species, the overall deposition of this complement component on all tested mucormycete species opsonized with NHS was measured to be moderate (Figure 1A), which was surprising since this part of the complement system is activated especially via antigen-bound immunoglobulins [14]. Considering that, normally, there should not be any specific antibodies against mucormycetes in the used NHS pool (due to the low prevalence in Europe [30]), the detected C1q deposition could be due to pentraxin 3 binding to the surface of the spores, thereby, activating the classical and the lectin pathways [31]. Binding of pentraxin 3 to spores, and, thus, exerting non-redundant functions, was already described for *Aspergillus fumigatus* [32,33].

While the activation of the classical pathway by mucormycete spores was moderate overall when opsonized with NHS (Figure 1A), the differences in C1q deposition were more pronounced between the spores of the tested mucormycetes opsonized with normal mouse serum as a source of complement (Figure 1D). Variations in pathway activation routes between humans and mice [34] can also explain the significant difference in C3c deposition on spores of *M. circinelloides* species opsonized with either NHS (Figure 1B) or NMS (Figure 1E). The binding patterns of human C1q (Figure 1A) and C3c (Figure 1B) on mucormycete spores differed significantly from each other, which could be due to the alternative pathway participating to a higher extent than the classical pathway. In contrast, the murine binding patterns of C1q (Figure 1D) and C3c (Figure 1E) looked the same, indicating that the classical pathway could be the main driver for the C3c deposition.

In vivo, we studied the relevance of two different elements of the immune system, namely, platelets and the complement system, on the pathogenesis of disseminated mucormycosis in comparison to neutropenia, which is a known risk factor for this devastating disease.

In addition to forming thrombi after an injury, thereby, maintaining homeostasis in the circulatory system, platelets are versatile players in the immune continuum and react to various pathogens [15,16]. Fungal pathogens such as *Aspergillus fumigatus* [35,36,37] and different mucormycetes [21] are detected by platelets, and antifungal effects are executed. For *Aspergillus fumigatus,* these effects were shown to be essential in vivo for survival during a pulmonary infection [38]. For mucormycetes, platelets were demonstrated to adhere in vitro to both spores and hyphae of clinical isolates of *Rhizopus*, *Lichtheimia*, *Rhizomucor*, and *Mucor* species; upon contact, hyphal growth was diminished, and damage was inflicted on hyphae [21]. In contrast to these promising findings, our study indicated that in vivo platelets are not crucial for the outcome in case of a disseminated mucormycosis (Figure 4A–F). An unexpected effect of thrombocytopenia was depicted as a tendency towards a reduced fungal load in the kidneys of thrombocytopenic mice compared to immunocompetent animals when infected with *R. microsporus*, *L. ramosa*, or *M. circinelloides* (Figure 5). This might point towards a possible participation of platelets in the dissemination of these isolates. Upon activation, platelets release pro-inflammatory microparticles, thereby, permeabilizing blood vessels for the migration of immune cells [39,40], but this permeabilization might as well be exploited by spores to disseminate into tissues. Furthermore, platelets adhered to spores can facilitate the attachment of those spores to endothelial cells due to CD154 on platelets, which is a ligand for the CD40 on endothelial cells [15,39]. Since a beneficial outcome of platelet-depleted mice was not observed, this effect on the spore distribution has to either be subtle or counteracted by other negative effects caused by the thrombocytopenia such as prolonged bleeding upon angioinvasion. Another observation in thrombocytopenic mice challenged with *R. microsporus* or *M. circinelloides* was the significantly increased platelet activation of the remaining platelets compared to in immunocompetent animals (Figure 6). This might lead to an increase in local thrombotic events in these mice as a pathological feature of mucormycosis, which might counterbalance the decreased fungal burden in the kidneys.

The complement system exerts a broad spectrum of functions in immune surveillance, homeostasis, and tissue repair [41], one of which revolves around fragments of the central C3 (C3b, iC3b, C3c). Those complement fragments act as opsonizing molecules and stimulate the internalization and, therefore, the elimination of pathogens by interacting with receptors on phagocytes [13]. Although Granja and coworkers showed in vitro that less C3 was deposited on hyphae of *M. polymorphosporus* than on spores [42], we found in vitro that C3c deposition on spores (Figure 3) still negatively correlates with the virulence in vivo (Figure 2). We hypothesize that poor C3c deposition, and, consequently, worse recognition and clearance by phagocytes, plays a role in the augmented virulence of *R. arrhizus* and *R. microsporus*. Surprisingly, even though *Rh. pusillus* showed only minor amounts of C3c on the spore surface, the tested isolate exhibited a low virulence in immunocompetent mice. Nevertheless, the complement system seems to play an important role in fighting off *Rh. pusillus*, since C3- and C6-deficient animals infected with 5 × 10^5^ spores of *Rh. pusillus* had a significantly worse outcome compared to immunocompetent animals (Figure 4E). The survival curves indicate that C6 is a key player in *Rh. pusillus* infections, even more significant than neutrophilic granulocytes. This points towards the importance of other effects of the complement system, such as indirectly luring phagocytes to the site of infection by anaphylatoxins or via effects of the C5b-9 complex. In its soluble form, C5b-9 complex shows several immunological functions such as recruiting polymorphonuclear leukocytes (PMN) to the site of infection, promoting transendothelial migration of PMNs, and induction of endothelial permeabilization [43]. As the membrane attack complex (MAC), the C5b-9 complex integrates into the cell membrane, subsequently, leading to the osmotic lysis of the cell [14,44].

Although the formation of the MAC, which establishes a pore 11 nm in diameter and 30 nm in length [45], and its role in the destruction of hyphae and spores need to be determined, mucormycetes could be more prone to be affected by the MAC compared to other pathogenic filamentous fungi such as *Aspergillus fumigatus.* Mucormycetes differ from *Aspergillus fumigatus* and other higher filamentous fungi as they have scarcely septate hyphae and fewer glucans, but more chitin and heteropolymers of higher solubility, in their cell wall [46,47]. These heteropolymers, mainly fucose and glucuronic acid, are not tightly interlinked and could facilitate the incorporation of the MAC into the cell membrane. Furthermore, the reduced hyphal cell wall thickness from 50 nm to 500 nm in *R. sexualis* and *R. homothallicus*, depending on the age of these fungal species [48], and from 40 nm to 60 nm in *M. rouxii* [49], compared to the thick cell wall of *Aspergillus fumigatus*, which varies from 85 nm to 315 nm [50], could render mucormycetes more prone to pore formation. In the case of pore formation, the fungus is killed even more easily since these fungi form non- or pauci-septate hyphae and, therefore, are not well protected against damages induced to their cell membrane [46].

The high virulence of *R. arrhizus* (Figure 2) was not reflected by the fungal load in the kidneys of immunocompetent mice (Figure 5). This discrepancy between the fungal burden and virulence was also observed in another study [26]. This fact might result from the early death of the animals with shorter fungal growth and, therefore, decreased fungal DNA in the organs. Hence, we can rule out fungal burden in the organs as the cause of reaching the humane endpoint, but rather it is a cytokine storm syndrome [51] and/or massive thrombosis due to the angioinvasion, which would fit with the highest platelet activation in immunocompetent animals (Figure 6).

In conclusion, our results provide strong evidence that complement, in contrast to platelets, plays a central role in fighting off mucormycetes. The complement system could play an indirect role by recruiting phagocytes and other immune cells to the site of infection, inducing endothelial permeabilization and promoting cytokine production by either anaphylatoxins, C3a and C5a, or soluble C5b-9 complex [14,43]. Complement might also act directly on mucormycetes by promoting phagocytosis via opsonization or leading to osmotic lysis caused by the pore formation of the membrane attack complex (MAC) [13,14].

Based on our findings, the next step is to study the exact mechanisms behind the role of the complement system in fighting off mucormycetes. In particular, to what extent the MAC is able to penetrate and subsequently harm these scarcely septate fungi. A better understanding of how the complement system affects the defense against mucormycetes is a prerequisite for the development of novel, complement-based adjunctive therapies.

## Data Availability

Not applicable.

## Supplementary Materials

The following supporting information can be downloaded at: https://www.mdpi.com/article/10.3390/jof9020162/s1, Table S1: Mucormycete strains used in this study.
