# Supplementary material for: Complement, but Not Platelets, Plays a Pivotal Role in the Outcome of Mucormycosis In Vivo
Source: J Fungi (Basel). 2023 Jan 25;9(2):162. doi: 10.3390/jof9020162 (PMC9965864; doi:10.3390/jof9020162)
Supplement: Supplementary file 1 [file jof-09-00162-s001.zip › jof-2150104-supplementary.pdf]

Table S1: **Mucormycete strains used in this study.**

species	internal IDs	CBS number	origin of isolation	sequenced
Lichtheimia corymbifera	LCJ2; J1; L1	CBS 429.75	soil, Afghanistan	yes
	LCJ3; LC4; LC9	CBS 109940	human finger, tissue, Norway	yes
	LCJ4	CBS 120581	human bronchia, France	no
	LCJ6; IBML 4 – M 10012	n/a	cattle, gut	no
	LCJ7; IBML 5 – M 10005	n/a	horse, gut	no
	LCJ8; IBML 6 – D 10005	n/a	horse, gut	no
	LCJ9; CNM – CM 3415	n/a	human, ear swab	no
	LCJ10; CNM – CM 5039	n/a	human, peritoneal drainage	no
	LCJ11; P. O. 623	n/a	stork, lung	no
	LCJ12; P. O. 829	n/a	stork, lung	no
	LCJ13; P. O. 612 B	n/a	stork, lung	no
	LCJ14; P. O. 909 B	n/a	stork, lung	no
	LCJ15	CBS 102.48	environment, moldy shoe	no
	LCJ16	CBS 115811	environment, indoor air	no
	LCJ17	n/a	human, scale	no
	LC1; AS10	n/a	n/a	yes
	LC2; AS19	n/a	n/a	yes
	LC3; AS61	n/a	n/a	yes
	LC6	n/a	n/a	no
Lichtheimia ramosa	LRJ1; LR5	CBS 101.55	human cornea	yes
	LRJ6; IBML1-D10007	n/a	n/a	no
	LRJ7	CBS 270.65	n/a	no
	LRJ11; CNM-CM 5396	n/a	n/a	no
	LRJ12	n/a	n/a	no
	LRJ13; CNM-CM 5399	n/a	n/a	no
	LRJ14; CNM-CM 3013	n/a	n/a	no
	LRJ15	n/a	n/a	no
	LRJ19	CBS 271.65	n/a	no
	LRJ25	CBS 100.55	n/a	no
	LRJ26	n/a	n/a	no
	LRJ27	n/a	n/a	no
	LRJ28; CNM-CM 5111	n/a	n/a	no
	LRJ29; P.O. 905A	n/a	n/a	no
	LRJ30; P.O. 909A	n/a	n/a	no
	LRJ31	n/a	n/a	no
	LRJ33	n/a	n/a	no
	LR1; S29	n/a	n/a	yes
	LR2; AS35	n/a	patient isolate	yes
	LR3; AS76	n/a	n/a	yes
Rhizopus microsporus	RM1; RM9; AS54	CBS 102277	patient isolate, rhinocerebral infection	yes
	RM3; F33	n/a	n/a	yes
	RM4; AS73	n/a	patient isolate	yes
	RM5; 75-10	n/a	n/a	yes
	RM6; 30-10	n/a	n/a	yes
Rhizopus arrhizus	RO1; F2	n/a	n/a	no
	RO6; 44-12	n/a	patient isolate, tracheal secrete	yes
	RO7; R2	CBS 126971	patient isolate	yes
Rhizomucor pusillus	RmP1; F12	n/a	n/a	yes
	RmP3; AS16	n/a	n/a	yes
	RmP5; RP3	CBS 219.31	pig, kidney	no
Mucor circinelloides	M2; S44	n/a	patient isolate	yes
	M4; 39-10	n/a	patient isolate, skin swab	yes
	M6; MC6	CBS 394.68	beef meat	yes
